# Supplementary material for: Etiology and pattern of maxillofacial trauma
Source: PLoS One. 2022 Sep 29;17(9):e0275515. doi: 10.1371/journal.pone.0275515 (PMC9522305; doi:10.1371/journal.pone.0275515)
Supplement: Attachment — Submitted filename: Response to reviewer comments.docx [file pone.0275515.s001.docx]

**Response to reviewer comments**

**We appreciate the comments of reviewers and modified the article concerning their suggestions**

Reviewer #1: - In your study titled 'ETIOLOGY AND PATTERN OF MAXILLOFACIAL TRAUMA-REGRESSION ANALYSIS', 'regression analysis' is a common analysis method. Therefore, I recommend using the title as follows, excluding that analysis. 'ETIOLOGY AND PATTERN OF MAXILLOFACIAL TRAUMA'

Reply: We changed the title as suggested.

- INTRODUCTION
The content of the last paragraph of the introduction is very similar to that of Objectives. Therefore, we recommend that you delete Objectives or describe it in a different content from the previous paragraph.

Reply. It has been modified as suggested

- MATERIALS AND METHODS:
In your study, the study subjects were limited to patients who visited the hospital for 2 weeks,
It is thought that the patients selected for the study were followed up from January 2019 to June 2021.
However, this part is not fully understood with the research method described by the authors. Therefore, to help the reader understand, please amend the following sentence to describe the content more accurately.

Reply: It has been amended as suggested.

'All the patients (including both genders) between age 6-60 years presenting within 2 weeks of trauma to oral and maxillofacial unitwere included in this study'
'this descriptive study was conducted in the Department of Oral and Maxillofacial Surgery, Lady Reading Hospital Peshawar from Jan 2019 to June 2021.'

Also, in the title, regression analysis was mentioned as an analysis method, but the content was omitted in the research method.
Reply: It has been readdressed as suggested.

- RESULTS
Currently table 1 is less readable. Authors are advised to put effort into presenting the table in a more neatly organized way to increase the readability of the reader.
Also, in presenting univariate results, rather than explaining each content in detail by dividing it into gender, age, and cause, detailed results are presented using tables, and in the case of content explained in writing, more impactful content is presented. I think it would be preferable.
Reply: It has been readdressed as suggested.

- DISCUSSION
Is it correct to consider only Gender as 'Cultural and socioeconomic factors' in your study?
Although gender is a factor that includes a relatively social concept when compared with sex, it seems unreasonable to think that 'Cultural and socioeconomic factor' is considered only with gender.
Considering that it is an etiology study, we recommend that you add analysis by considering more diverse variables.

Reply: It has been readdressed as suggested.

Reviewer #2: I would just like to point out a few things:
1. The Materials and Methods section on the Abstract is insufficient.There should be a sentence or two about how the data was collected and analyzed.

Reply: It has been readdressed as suggested.
2. In the Introduction section I would recommend that the Objective be merged with the last paragraph and not written as a separate section.

Reply: It has been readdressed as suggested.
3. There are a few grammatical errors that require correction throughout the manuscript.

Reply: We have thoroughly revised the manuscript as suggested

6. PLOS authors have the option to publish the peer review history of their article ([what does this mean?](https://journals.plos.org/plosone/s/editorial-and-peer-review-process#loc-peer-review-history)). If published, this will include your full peer review and any attached files.
